# Implication of Microsatellite Instability Pathway in Outcome of Colon Cancer in Moroccan Population

**DOI:** 10.1155/2019/3210710

**Published:** 2019-12-07

**Authors:** Fatima El Agy, Ihssane El Otmani, Asmae Mazti, Nada Lahmidani, Abdelmalek Oussaden, Mohamed El Abkari, El Bachir Benjelloun, Wadih Moukit, Hicham El Bouhaddouti, Imane Toughrai, Karim Ibn Majdoub Hassani, Khalid Maazaz, Zineb Benbrahim, Nawfal Mellas, Karima El Rhazi, Karim Ouldim, Sanae El Bardai, Sidhi Adil Ibrahimi, Khalid Ait Taleb, Sanae Bennis, Chbani Laila

**Affiliations:** ^1^Medical Center of Biomedical and Translational Research, Hassan II University Hospital, Fez, Morocco; ^2^Department of Pathological Anatomy, Hassan II University Hospital, Fez, Morocco; ^3^Department of Gastroenterology, Hassan II University Hospital, Fez, Morocco; ^4^Department of Visceral Surgery, Hassan II University Hospital, Fez, Morocco; ^5^Department of Obstetrics and Gynecology, Military Training Hospital Mohammed V, Rabat, Morocco; ^6^Department of Oncology, Hassan II University Hospital, Fez, Morocco; ^7^Laboratory of Epidemiology, Faculty of Medicine and Pharmacy, Fez, Morocco; ^8^Department of Medical Genetics and Ontogenetic, Hassan II University Hospital, Fez, Morocco; ^9^Department of General Surgery, Hassan II University Hospital, Fez, Morocco; ^10^Biomedical and Translational Research Laboratory, Faculty of Medicine and Pharmacy, Morocco

## Abstract

**Background:**

Tumors with microsatellite instability (MSI tumors) have distinct clinicopathological features. However, the relation between these tumor subtypes and survival in colon cancer remains controversial. The aim of this study was to evaluate the overall survival (OS) in patients with MSI phenotype, in FES population.

**Methods:**

The expression of MMR proteins was evaluated by immunohistochemistry for 330 patients. *BRAF*, *KRAS*, and *NRAS* mutations were examined by Sanger sequencing and pyrosequencing methods. The association of MSI status with a patient's survival was assessed by the Kaplan–Meier method and log-rank test.

**Results:**

The mean age was 54.6 years (range of 19-90 years). The MSI status was found in 11.2% of our population. MSI tumors were significantly associated with male gender, younger patients, stage I-II, right localization, and a lower rate of lymph node and distant metastasis. The OS tends to be longer in MSI tumors than MSS tumors (109.71 versus 74.08), with a difference close to significance (*P* = 0.05).

**Conclusion:**

Our study demonstrates that MSI tumors have a particular clinicopathological features. The results of survival analysis indicate that the MSI status was not predictive of improved overall survival in our context with a lower statistical significance (*P* = 0.05) after multivariate analysis.

## 1. Introduction

Microsatellite instability (MSI) or mismatch repair deficient (dMMR) is one of the main pathogenetic pathways leading to the development of colorectal cancer [1]. MSI phenotype is due to dysfunction of a DNA mismatch repair (MMR) system in microsatellite replication [2]. The MMR system is composed of four MMR genes and their encoded proteins (MLH1, MSH2, MSH6, and PMS2) [3]. These proteins form heterodimers that repair DNA damage (mismatches as well as short insertion or deletion loops) [4]. In hereditary nonpolyposis CCRs (e.g., Lynch syndrome), 90% of the MMR alterations are mainly due to constitutional mutations of the MLH1 and MSH2 gene (more rarely MSH6 or PMS2 gene) or alteration in EPCAM (TACSTD1) gene that causes epigenetic silencing of MSH2 [5, 6], while 10 to 15% of all sporadic CCRs are due to hypermethylation of CpG islands in the MLH1 promoter [6].

Several studies have shown that dMMR tumors have special clinicopathological features, including poor differentiation, right colon location, abundant tumor-infiltrating lymphocytes, and mucinous histology [7]. Furthermore, these tumors are associated with the presence of BRAF mutations [8]. MMR tumors are diagnosed in 15-20% of localized CCRs, especially in stage II, although they represent 3-5% of metastatic CCRs [9].

Variable results are reported in the literature, about the association between MSI status and survival in colorectal cancer. Tumors diagnosed at stage II or III with MSI have better prognosis than MSS tumors [10, 11]. In addition, many studies have demonstrated that CCRs with MSI status showed poorer response to 5-fluorouracil (5FU) compared to CCRs with MSS status [10]. For patients with stage III and MSI, it has been confirmed that only those with suspected germinal mutations can benefit from treatment with 5FU [11].

In the Moroccan population, few studies have reported the prognostic factors of MSI colon cancer. Therefore, in this study, we aimed to assess the frequency of loss in MMR protein expression, to compare the clinical and pathological features of MSI versus MSS colon cancers, and to evaluate the survival rates in patients with MSI tumors in association with other clinicopathological features, for the first time in the FES population.

## 2. Materials and Methods

### 2.1. Ethics

This study protocol was reviewed and approved by Hassan II University Hospital Ethics Committee of FEZ, Morocco, under reference no. 13/18. All patients gave informed consent before the start of the study.

**Figure 1:**
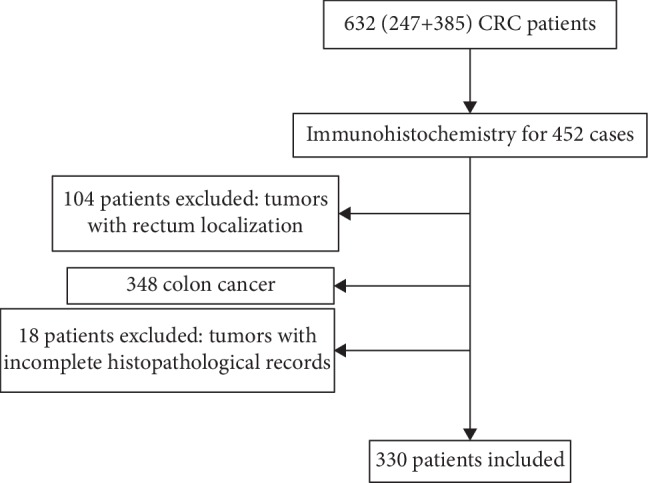
Flow chart of a patient's enrollment.

A total of 330 patients diagnosed with colon cancer were included in this study, in the Department of Pathology of Hassan II University Hospital, Fez, Morocco, from 2013 to 2019. Medical charts have been reviewed, and patients have been selected using the following selection criteria: (a) patients had histologically confirmed primary adenocarcinoma (b) all cases with pathologic stage I-IV colon cancer and underwent surgical resection for CC tumor. However, patients were excluded if their records were incomplete and without histological confirmation of colon adenocarcinoma and if they had rectal cancer (Figure 1).

The clinical and pathological data including age, gender, main histological pattern, tumor grade, tumor stage, numbers of dissected regional lymph nodes, family history of colon cancer, follow-up, and outcome have been obtained from the patient's medical records and pathology reports.

### 2.2. Identification of HNCCP Patients

Family history and clinical data were reviewed to determine patients who fulfilled the Amsterdam (I-II) criteria and met the Bethesda guidelines for molecular Lynch syndrome (LS) testing. However, any patient did fulfill the clinical features concerning for LS.

### 2.3. Detection of MMR Protein Expression by Immunohistochemistry

The mismatch repair tumor status (MSS or MSI) was assessed by immunohistochemistry (IHC) to detect the intact or the loss expression of the MMR proteins (MLH1, PMS2, MSH2, and MSH6).

The IHC study was assessed on unstained formalin-fixed paraffin-embedded (FFPE) tumor tissue sections of 5 *μ*m thickness, on the automated immunostainer Ventana Benchmark ULTRA. We have employed monoclonal antibodies specific for each MMR protein, MLH1 (G168-728/CELL MARQUE), MSH2 (G219-1129/CELL MARQUE), MSH6 (44/CELL MARQUE), and PMS2 (MRQ-28/CELL MARQUE). Adjacent normal tissue (lymphocytes or normal glandular cells) was used as an internal control for positive staining (should always show staining).

### 2.4. Full RAS and BRAF Mutation Analysis

#### 2.4.1. Genomic DNA Extraction

Tumoral DNA was extracted from paraffin-embedded tumor sections. The blocks with higher proportion of tumors cells were selected by a pathologist on hematoxylin-, safran-, and eosin-stained slides. From the selected FFPE tumor block, 4–8 sections of 5 *μ*m thickness were obtained for DNA extraction using the QIAamp DNA FFPE Tissue Kit (Invitrogen), according to the manufacturer's instructions.

#### 2.4.2. BRAF Mutation Analysis

BRAF testing was performed for 200 patents, using Sanger sequencing to distinguish sporadic dMMR CC that exhibits the V600E mutation in the BRAF oncogene. DNA was amplified using PCR (Master Mix (2X) kits) according to the manufacturer's protocols. The purified PCR products were sequenced using the direct sequencing with BigDye Terminator V3.1 Cycle Sequencing Kit (ABI Prism) and analyzed on Applied Biosystems 3500Dx Genetic Analyzer (Applied Biosystems).

#### 2.4.3. RAS Mutation Analysis

KRAS and NRAS molecular testing was performed using Sanger sequencing and pyrosequencing methods. Pyrosequencing was performed on the Qiagen PyroMark Q24 device according to the CE-IVD-marked therascreen RAS Pyro Kit Handbook. Sanger sequencing was performed as described previously (BRAF mutation testing).

### 2.5. Statistical Analysis

Statistical analysis was performed using IBM SPSS Version 20.0. The associations between the clinicopathological features of tumors and the microsatellite status (MSI/MSS) were evaluated using a chi-square test or Fisher exact tests. Tests were statistically significant when *P* < 0.05. An unpaired t-test with Welch's correction was used to analyze continuous data. Survival rates were analyzed using the Kaplan–Meier method, and survival curves were compared with the log-rank test. Analysis was performed using a Cox proportional hazard model to identify prognostic factors. Factors that were significant in univariate analysis were included in the multivariate model.

## 3. Results

### 3.1. Patient Characteristics

The mean age was 54.62 years (range of 16-90 years). There was a slight predominance of male gender with 174 (52.7%) men and 156 (47.3%) women patients. The left colon was the most frequent site of CC in our population (*n* = 254, 77%), while the right colon was diagnosed in 76 cases (23%). Adenocarcinoma was the main histological type of CC in our study with 278 (84.2%) cases. The moderate and the well differentiation grades were found, respectively, in 128 (38.9%) cases and 173 (52.6%) cases, despite the fact that only 28 (8.5%) cases were poorly differentiated. Regarding the pathologic stage of tumors, 161 (48.8%) were stage II, 73 (22.1%) were stage III, 68 (20.6%) were stage IV, and 28 were stage I (8.5%). 68 (20.6%) patients were classified as the metastatic group. All clinicopathological features of patients are summarized in Table 1.

**Figure 2:**
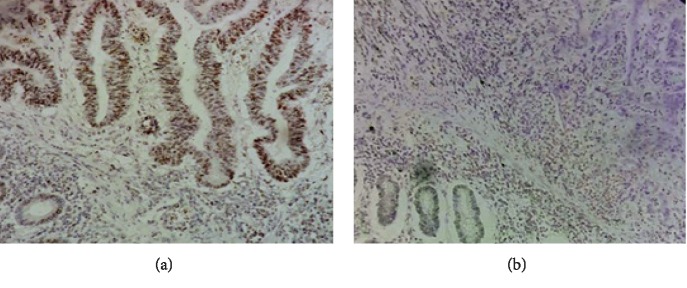
Immunohistochemical staining for MMR proteins. (a) Overexpression of MLH1 protein in colon adenocarcinoma (magnification 200x). (b) MLH1 loss in colon adenocarcinoma (magnification 200x).

Among the total of 330 colon cancer (CC) patients who prospectively tested for microsatellite instability during the study period, 293 were MSS (88.8%) and 37 MSI (11.2%) (Figure 2). Of the 37 MSI tumors, the loss of expression of MMR proteins was as follows: MLH1/PMS2 (*n* = 18) (5.4%), MSH2/MSH6 (*n* = 11) (3.3%), MSH6 (*n* = 1) (0.3%), PMS2 (*n* = 3) (1%), and MLH1 (*n* = 4) (1.3%).

### 3.2. BRAF, KRAS, and NRAS Analyses

#### 3.2.1. BRAF Analysis

BRAF V600E mutation testing was performed on all patients with MSI tumors (37 patients) and on 163 patients with MSS tumors, while we did not find any case with both *MLH1* protein expression loss and BRAF mutation.

#### 3.2.2. KRAS and NRAS Analyses

**Figure 3:**
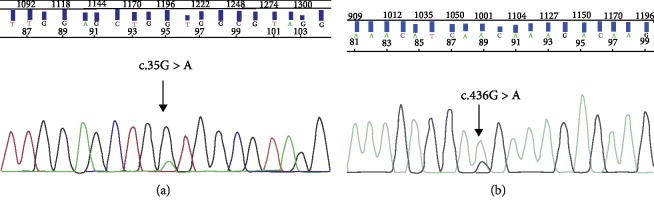
Direct sequencing chromatograms. (a) *KRAS* exon 2 mutation (c.35G>A, p.G12D change), (b) *KRAS* exon 4 mutation (c.436 G>A, p.A146T change).

RAS analysis was performed in 116 patients. We detected KRAS exon 2 mutations in 36 patients (33.3%). Among the 80 patients with KRAS exon 2 wild-type (69.0%), we identified two mutations in KRAS exon 4 (codon 146 (1.7%)). However, no mutation was detected in the NRAS gene (0%) (Figure 3).

### 3.3. Pathological Features of MSI Tumors

A summary of the main clinicopathological features of MSI cancers compared to MSS cancers is shown in Table 2. In the majority of cases, tumors with MSI were located in the right colon compared to MSS tumors (*P* ≤ 0.001). Male gender was significantly associated with MSI tumors (72.0% versus 28.0%, *P* = 0.04). Moreover, MSI tumors were more commonly found in patients under 57 years (*P* = 0.05). As for distant metastases, no patient with MSI status had distant metastases at the time of diagnostic, and the difference was statistically significant (*P* = 0.03). Regarding pathologic disease stage, stage I-II were more common in MSI tumors (*P* = 0.02). Histologically, the degree of differentiation was associated with MSI tumors and the difference was statistically significant (*P* = 0.04). In contrast, no significant correlation was found between MSI tumors and histologic subtype, T stage, vascular invasion, perineural invasion, family history of colon cancer, and RAS mutation.

### 3.4. Nodal Counts in Stage I-IV Colon Cancer: Comparison of the Total Lymph Nodes (LNs), Metastatic LNs, and LNR according to MSI Status

When analyzing our cohort, MSI tumors were associated with a significantly higher total LN count (mean: 23.440 vs. 19.075, *P* = 0.04). However, the number of positive LNs was significantly lower in MSI patients (mean: 0.28 vs. 1.34, *P* = 0.007). The LNR was also lower in MSI tumors, and the difference was statistically significant (0.01 vs. 0.08, *P* = 0.01). The lymph node characteristics of MSI tumors are compiled in Table 3.

When analyzing our cohort, MSI tumors were associated with a significantly higher total LN count (mean: 21.08 vs. 17.68, *P* = 0.04). However, the number of positive LNs was significantly lower in MSI patients (mean: 0.62 vs. 1.33, *P* = 0.03). The LNR was also lower in MSI tumors, and the difference was statistically significant (0.03 vs. 0.08, *P* = 0.02). The lymph node characteristics of MSI tumors are compiled in Table 3.

### 3.5. Clinicopathological Features of Patients with Right Colon Cancer and Left Colon Cancer

Table 4 shows the basic characteristics between the right colon cancer (RCC) and the left colon cancer (LCC). The mean age of right-sided tumors was significantly younger than that of left-sided tumors (52.5 and 58.5 years old, respectively, *P* = 0.01). Patients with RCC were significantly more likely to be male in our study (*P* = 0.03). Regarding pathologic grade, moderate differentiated tumors were less common in RCC than in LCC patients (*P* = 0.05). In addition, a mucinous adenocarcinoma subtype was significantly associated with RCC tumors than with LCC tumors (*P* = 0.01). At all stages, the mean of harvested lymph nodes was significantly higher in RC tumors than in LCC tumors (22.2 vs. 18.17, *P* = 0.01). More LCC had a higher incidence of metastatic disease at diagnosis (15.8% vs. 6.3%, *P* = 0.05). In this study, 30.2% of RCC patients were microsatellite unstable compared to 5.9% of LCC patients (*P* ≤ 0.001). The frequency of RAS mutation did not differ between RCC tumors and LCC tumors in our study.

### 3.6. Comparison of Clinicopathological Features between Patients Less than 58 Years Old and Older

As shown in Table 5, we divided the patients into two groups: patients who were 57 years old and less and patients more than 57 years old. The proportion of right colon cancers was higher in patients 57 years old and less than in older patients (*P* = 0.02). The proportion of poorly differentiated tumors was higher in the youngest age subgroup than in the oldest (11.8% vs. 5.0%, *P* = 0.05). Interestingly, the presence of a family history of colon cancer was significantly associated with the youngest age subgroup (*P* = 0.02). The youngest age subgroup had more nodes examined than the oldest age subgroup (*P* < 0.001). Moreover, 90.7% of the youngest patients have more than 12 lymph nodes collected, compared with 80.6% in the oldest patients (*P* = 0.04). Generally, the MSI status was also significantly correlated with the youngest age subgroup in our series (*P* = 0.01).

## 4. Survival Analysis

The median length of the follow-up period was 30.00 months (range 0-117 months). From 330 patients' colon cancer, 33 patients (10%) developed distant metastasis during the follow-up period. The time to metastasis ranged from 2 to 46 months (median = 11.00 months). The most common metastatic site was peritoneum (*n* = 13, 43.3%) followed by the lung (*n* = 11, 36.6%), liver (*n* = 10, 33.3%), Os (*n* = 1, 3.3%), ovary (*n* = 1, 3.3%), distant lymph nodes (*n* = 1, 3.3%), and head (*n* = 1, 3.3%). Regarding recidivism, 9 of 330 patients developed a disease relapse. The median length of recidivism was 11.0 months (range 0-29 months).

**Figure 4:**
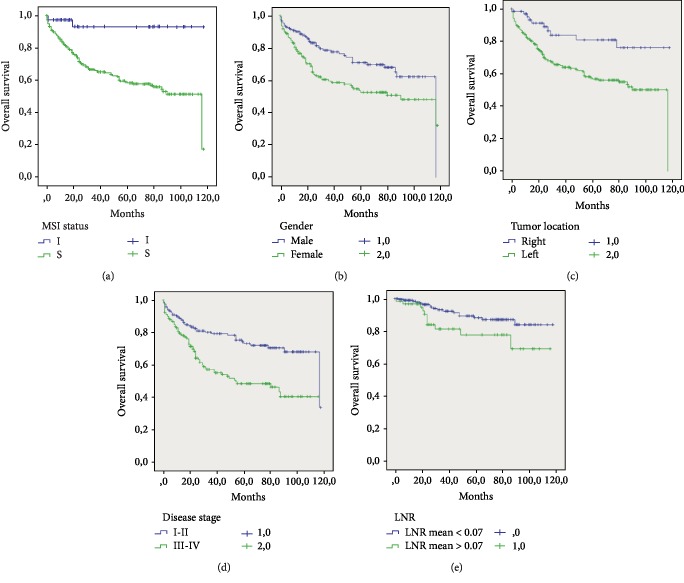
The Kaplan–Meier overall survival curve of the 330 colon cancer patients according to different variables: (a) MSI status, (b) Gender, (c) tumor localization, (d) disease stage, and (e) LNR.

According to our findings in our cohort, we observed a better prognosis of MSI patients compared with that of MSS patients and the difference was statistically significant (109.71% vs. 74.08%, *P* = 0.001) (Figure 4(a)). The variables associated with the OS are shown in Table 6. On univariate analysis, the male gender was significantly associated with OS (*P* = 0.003) (Figure 4(b)). The OS of patients with stage III-IV disease was significantly lower than that of patients with stage I-II disease (63.85% vs. 89.48%, *P* = 0.000) (Figure 4(d)). Moreover, right localization and male gender were found to be statistically significant predictors of poor outcomes in our population (Figure 4(c)). Interestingly, we stratified the LNR into 2 subgroups based on the mean value of 0.07, and we documented that the higher value of LNR was a better predictor of prognosis (*P* = 0.03) (Figure 4(e)).

Cox proportional hazard regression was performed for factors that were significant in univariate analysis (*P* < 0.05) (MSI status, gender, tumor site, disease stage, and LNR). Multivariate analysis revealed that female gender, left localization, and III-IV were the independent poor prognostic factors for OS in colon cancer Table 6.

## 5. Discussion

In the present study, we aimed to evaluate the impact of MSI status and clinicopathological features on overall survival in the Moroccan population. Our findings suggest that MSI tumors occur in 11.2% in the FES population which is similar to previous studies which confirmed that the rate of dMMR tumors in colon cancer is between 10% and 15% [12, 13]. In addition, we observed that MSI tumors were commonly found in younger patients (≤57) and in the right colon as reported in the literature [13, 14]. In line with other studies [10, 14, 15], we found that MSI tumors were mostly diagnosed in stage II (75.7%). This finding could explain the more favorable prognosis of MSI subtype, described in several studies [14, 16].

Our study has been able to demonstrate a positive relationship between MSI tumors and total lymph node count. Recently, Tian et al. [13] found the same observation in a cohort of 1250 stage II-IV CRC cases. These findings are a possible explanation for the high immune response observed in MSI tumors [17, 18]. In addition, the patients enrolled in our study showed a significantly lower rate of positive lymph node. The low lymph node ratio (LNR) was also associated with MSI tumors in all stages of CC. This finding is in line with the results reported by Ghanipour et al. [15] in a recent publication which indicated a relevant association between MSI high and low rate of LNR. A previous study documented that low LNR is a predictor of good outcome [19, 20]. These results explain the better survival of patients with MSI status. Despite better outcomes, our study showed a positive correlation between MSI status and poor differentiation, which is reported as a factor of poor prognosis. This result is in agreement with several reports [21–23].

In our study, from 200 patients, we did not find any *BRAF* mutation in both MSI and MSS tumors. This result confirms the rarity of the V600E mutation in colon cancer, epically in the Moroccan population [17].

On the other hand, many investigators demonstrate that RCC and LCC show clinicopathological and molecular differences. In the present study, RCC tumors are more likely mucinous, commonly observed in younger patients, had less metastasis disease, and showed frequently MSI status. In univariate and multivariate analyses, we found that RCC had better OS than LCC and the significant difference of the Cox hazard ratio between the two subgroups was reported in our series (*P* = 0.05). Some studies have documented that overall survival is generally worse in the right colon compared to the left colon [24, 25], while others revealed no difference [18]. The molecular profile of right colon cancer characterized by the presence of RAS/BRAF mutation which correlated with worse outcome [26]. In the present study, there was no association between RC location and RAS mutation.

The relationship between MSI tumors and OS is unclear according to Elias et al. [19]. In our study, there was an improved OS in patients with stage I-IV and MSI status. In line with our findings, previous studies have demonstrated that MSI tumors have a better clinical outcome [27]. The prognosis value of MSI tumors could be explained by the low rate of metastasis at diagnosis observed in these tumors, which is confirmed in our study. Indeed, Ferri et al. [21] have reported that the MSI patients with locally advanced colorectal cancers had a better prognosis.

According to many studies, low LNR was proven to be a strong prognostic factor of survival [20, 26]. In addition, Emterling et al. [22] documented a significant association between higher LNR and reduced OS and time to recurrence in patients with stage III of CRC. In our study, we found an improved OS in patients with low LNR, with a significant statistical difference on univariate analysis (*P* = 0.03), although TLN was not found to be an independent prognostic factor in our series on both univariate and multivariate analyses. In line with our results, Jass et al. [23] reported no significant difference of the Cox hazard ratio between OS and TLN.

Reviewing our data, we found that patients who were diagnosed with stage III-IV disease had the highest risk of death compared to those with stage I-II disease. Our results are in agreement with the finding recently reported by Weiss et al. [24]. Interestingly and contrary to previous studies [28, 29], we documented a strong significant correlation between male gender and OS in all stages, while other reports did not find any difference in survival between genders [30, 31]. Our result may be related to the proportions of colon cancer patients included in our study and the stronger correlation observed between MSI status and male gender. Moreover, the multivariate analysis revealed that MSI status, right localization, stage I-II, and male gender were the most significant prognostic factors for overall survival in Moroccan patients.

## 6. Conclusion

In summary, our study demonstrates that patients with MSI status have particular clinicopathological features like TLN, LNR, poor differentiation, right colon, locally advanced tumors, male gender, and younger patients. The results of survival analysis indicate that MSI status was not predictive of improved overall survival in our context with a lower statistical significance (*P* = 0.05). After cox regression analysis, the right localization of the tumor, I-II stage disease, and male gender showed a trend toward a better prognosis in our population.

## Figures and Tables

**Table 1:** Clinicopathological features of 330 patients.

Variables	*N* (%)
Age (mean)	54.62 years ± 14.7
<50	123 (37.3%)
>50	207 (62.7%)
Sex	
Female	156 (47.3%)
Male	174 (52.7%)
Tumor site	
Right colon	76 (23.0%)
Left colon	254 (77.0%)
Histologic subtype	
Adenocarcinoma	278 (84.2%)
Mucinous adenocarcinoma	29 (8.8%)
Others	23 (7%)
Histologic grade	
Well	173 (52.4%)
Moderate	128 (38.8%)
Poor	28 (8.5%)
Unknown	1 (0.3%)
Perineural invasion	
Yes	27 (8.2%)
No	272 (82.4%)
Unknown	31 (9.4%)
Vascular invasion	
Yes	39 (11.8%)
No	291 (88.2%)
Disease stage	
I	28 (8.5%)
II	161 (48.4%)
III	73 (22.1%)
IV	68 (20.6%)
MSI status	
MSS	293 (88.8%)
MSI	37 (11.2%)

*N*: number of cases; SD: standard deviation; MSS: microsatellite stable; MSI: microsatellite instability.

**Table 2:** Clinicopathological features of MSI cancers.

Characteristics	Defect MMR	Intact MMR	*P* value
Age			
Mean	51.5 (±14.18)	55.0 (±15.45)	0.1
≤57	26 (70.3%)	144 (49.1%)	**0.01**
≥57	11 (29.7%)	149 (50.9%)	
Gender			
Female	10 (27.0%)	146 (49.8%)	**0.007**
Male	27 (73.0%)	147 (50.2%)	
Tumor site			
Right colon	23 (62.2%)	53 (18.1%)	**0.000**
Left colon	14 (37.8%)	240 (81.9%)	
Histologic subtype			
Adenocarcinoma	30 (81.8%)	248 (84.6%)	
Mucinous adenocarcinoma	6 (16.2%)	23 (7.8%)	**0.1**
Others	1 (2.7%)	22 (7.5%)	
Histologic grade			
Well	15 (40.5%)	208 (71.5%)	**0.000**
Poor	22 (59.5%)	83 (28.5%)	
Vascular invasion			
Yes	7 (18.9%)	32 (10.9%)	**0.1**
No	30 (81.1%)	261 (89.1%)	
Perineural invasion			
Yes	2 (5.7%)	25 (9.5%)	**0.3**
No	33 (94.3%)	239 (90.5%)	
Family history of cancer			
Yes	3 (8.1%)	9 (3.1%)	**0.1**
No	34 (91.9%)	284 (96.9%)	
Tumor stage (T)			
T1	0 (0.0%)	4 (1.3%)	**0.5**
T2	5 (13.5%)	47 (16.2%)	
T3	25 (67.6%)	173 (59.5%)	
T4	7 (18.9%)	67 (23.0%)	
Distant metastases (M)			
M0	36 (97.3%)	222 (76.8%)	**0.001**
M1	1 (2.7%)	67 (23.2%)	
Disease stages			
I-II	28 (75.7%)	160 (54.6%)	**0.01**
III-IV	9 (24.3%)	133 (45.4%)	
RAS mutation			
Presence	6 (15.8%)	32 (84.2%)	**0.09**
Absence	12 (15.4%)	66 (84.6%)	

MMR: mismatch repair; SD: standard deviation.

**Table 3:** Comparison of lymph node features according to MSI status.

	MSI (*n* = 37)	MSS (*n* = 293)	*P* value (95% CI)
Total node			
Mean (SD)	21.08 (±9.94)	17.68 (±9.54)	*P* = 0.04 (0.053 to 6.74)
Positive node	0.62 (±1.49)	1.33 (±3.10)	*P* = 0.03 (-1.35 to -0.005)
Lymph node ratio	0.03 (±0.08)	0.08 (±0.27)	*P* = 0.02(-0.097 to -0.005)

MSI: microsatellite instability; MSS: microsatellite stable; SD: standard deviation.

**Table 4:** Comparison of clinicopathological characteristics between the right colon and left colon.

Characteristics	Right colon	Left colon	*P* value
Age			
Mean (SD)	51.4 (±15.7)	55.5 (±15.0)	**0.03**
≤57	47 (61.8%)	123 (48.4%)	**0.002**
≥57	29 (38.2%)	131 (51.6%)	
Gender			
Female	28 (36.8%)	128 (50.4%)	**0.02**
Male	48 (63.2%)	126 (49.6%)	
Histologic subtype			
Adenocarcinoma	59 (77.6%)	219 (86.2%)	**0.04**
Mucinous adenocarcinoma	12 (15.8%)	17 (6.7%)	
Others	5 (6.6%)	18 (7.1%)	
Histologic grade			
Well	42 (55.3%)	131 (51.8%)	**0.05**
Moderate	26 (34.2%)	102 (40.3%)	
Poor	8 (10.5%)	20 (7.9%)	
Vascular invasion			
Yes	14 (18.4%)	25 (9.8%)	**0.03**
No	62 (81.6%)	229 (90.2%)	
Perineural invasion			
Yes	7 (9.2%)	20 (9.0%)	**0.5**
No	69 (90.8%)	203 (91.0%)	
Total lymph node			
Mean (SD)	20.67 (±11.28)	17.28 (±8.85)	**0.01**
LN > 12	53 (74.7%)	82 (46.7%)	**0.000**
LN < 12	18 (25.3%)	105 (53.3%)	
Family history of cancer			
Yes	5 (6.6%)	7 (2.8%)	**0.1**
No	71 (93.4%)	247 (97.2%)	
Tumor stage (T)			
T1	1 (1.3%)	3 (1.2%)	**0.8**
T2	10 (13.2%)	42 (16.7%)	
T3	48 (63.2%)	150 (59.5%)	
T4	17 (22.4%)	57 (22.6%)	
Distant metastases (M)			
M0	68 (91.9%)	190 (75.4%)	**0.001**
M1	6 (8.1%)	68 (24.6%)	
Disease stages			
I	9 (11.8%)	19 (7.5%)	**0.002**
II	45 (59.2%)	116 (45.7%)	
III	17 (22.4%)	56 (22.0%)	
IV	5 (6.6%)	63 (24.8%)	
MSI status			
MSI	23 (30.3%)	14 (5.5%)	**0.0000**
MSS	53 (69.7%)	240 (94.5%)	
RAS mutation			
Presence	13 (34.2%)	25 (65.8%)	**0.4**
Absence	22 (28.2%)	56 (71.8%)	

SD: standard deviation; LN: lymph node; MSI: microsatellite instability; MSS: microsatellite stable.

**Table 5:** Comparison of clinicopathological parameters between patients less than 57 years old and older patients.

Characteristics	≤57	≥57	*P* value
Gender			
Female	88 (51.8%)	68 (42.5%)	**0.05**
Male	82 (48.2%)	92 (57.5%)	
Tumor site			
Right colon	47 (27.6%)	29 (18.1%)	**0.02**
Left colon	123 (72.4%)	131 (81.9%)	
Histologic subtype			
Adenocarcinoma	138 (81.2%)	140 (87.5%)	**0.07**
Mucinous adenocarcinoma	15 (8.8%)	14 (8.8%)	
Others	17 (10.0%)	6 (3.8%)	
Histologic grade			
Well	88 (52.1%)	85 (53.1%)	**0.05**
Moderate	61 (36.1%)	67 (41.9%)	
Poor	20 (11.8%)	8 (5.0%)	
Vascular invasion			
Yes	16 (9.4%)	23 (14.4%)	**0.1**
No	154 (90.6%)	137 (85.6%)	
Perineural invasion			
Yes	14 (9.1%)	13 (9.0%)	**0.5**
No	140 (90.9%)	132 (91.0%)	
Total lymph node			
Mean (SD)	24.5 (±10.30)	17.9 (±9.06)	**0.000**
LN > 12	68 (90.7%)	58 (80.6%)	**0.04**
LN < 12	7 (9.3%)	14 (19.4%)	
Family history of cancer			
Yes	9 (5.3%)	3 (1.9%)	**0.02**
No	161 (94.7%)	157 (98.1%)	
Tumor stage (T)			
T1	3 (0.0%)	1 (0.6%)	**0.4**
T2	22 (13.1%)	30 (18.8%)	
T3	101 (60.1%)	97 (60.6%)	
T4	42 (25.0%)	32 (20.0%)	
Distant metastases (M)			
M0	134 (79.8%)	124 (78.5%)	**0.4**
M1	34 (20.2%)	34 (21.5%)	
Disease stages			
I	13 (7.6%)	15 (9.4%)	**0.6**
II	80 (47.1%)	81 (50.6%)	
III	42 (24.7%)	31 (19.4%)	
IV	35 (20.6%)	33 (20.6%)	
MSI status			
MSI	26 (15.3%)	11 (6.9%)	**0.01**
MSS	144 (84.7%)	149 (93.1%)	
RAS mutation			
Presence	16 (46.8%)	22 (57.9%)	**0.3**
Absence	36 (42.1%)	41 (53.2%)	

SD: standard deviation; LN: lymph node; MSI: microsatellite instability; MSS: microsatellite stable.

**Table 6:** The clinical variables associated with overall survivals in the 330 colon cancer patients.

Variables	Univariate analysis		Multivariate analysis	
	Mean OS months (95% CI)	*P* value	Hazard ratio (95% CI)	*P* value
Age (yr)				
≥57	79.77 (71.86-87.68)	0.4		
<57	75.08 (66.59-83.56)			
Gender				
Female	69.09 (60.91-77.28)	0.003	1.52 (1.03-3.08)	0.03
Male	86.19 (78.37-94.01)			
Tumor site				
Right side	97.07 (85.97-108.17)	0.001	1.79 (0.98-3.71)	0.05
Left side	72.72 (66.27-79.16)			
Tumor stage				
I-II	89.48 (82.28-96.68)	0.000	1.49 (1.29-2.82)	0.001
III-IV	63.85 (55.48-72.22)			
LNR (mean)				
1	105.50 (100.36-110.64)	0.03	2.04 (0.80-5.18)	0.1
2	92.38 (80.66-104.10)			
TLN				
≥12	104.77 (96.26-113.27)	0.6		
<12	101.72 (95.73-107.71)			
Perineural invasion				
Yes	74.80 (58.63-90.96)	0.6		
No	82.64 (76.56-88.72)			
Vascular invasion				
Yes	88.47 (73.13-103.81)	0.1		
No	75.87 (69.73-82.02)			
MSI status				
MSI	109.71 (99.85-119.58)	0.001	0.11 (0.06-1.04)	0.05
MSS	74.08 (68.00-80.16)			
RAS mutation				
Presence	52.52 (40.918-64.133)	0.5		
Absence	56.75 (42.057-71.452)			

CI: confidence interval; OS: overall survival; LNR: lymph node ratio; TLN: total lymph node; MSI: microsatellite instability; MSS: microsatellite stable.

## Data Availability

The datasets used/or analyzed during the current study are available from the corresponding author on reasonable request.
